# Short-Term Dynamics of Behavioral Thermoregulation by Adults of the Grasshopper *Melanoplus sanguinipes*

**DOI:** 10.1673/031.007.2701

**Published:** 2007-05-04

**Authors:** Kevin M. O'Neill, Marni G. Rolston

**Affiliations:** ^1^ Department of Land Resources and Environmental Sciences, Montana State University, Bozeman MT 59717; ^2^ Department of Animal and Range Sciences, Montana State University, Bozeman MT 59717

**Keywords:** behavior, basking, orientation, habitat selection, thermal biology, thermal gradient, set point temperature, grazing

## Abstract

The short-term behavioral responses of adult grasshoppers, *Melanoplus sanguinipes* (F.) (Orthoptera: Acrididae), were examined after they experienced changes in microclimate when beingforced to change positions in their habitat. It was also determined if and when behavioral tactics allowed adults to achieve body temperatures within their preferred range. The preferred or set-point range, here taken as the interquartile range of temperatures selected on a laboratory thermal gradient, was estimated to be 37.4–40.5°C. In the field, adults progressed through a relatively consistent daily sequence of behaviors, basking on the soil early in the day, but moving onto vegetation as temperatures increased. Although basking allowed grasshoppers to maximize body temperature within the available range, as much as 7°C in excess of air temperature, they could not attain preferred body temperatures until soil surface temperatures reach about 35°C. Basking was more effective in grazed than ungrazed pastures due to a lower degree of shading of the soil surface. As soil surface temperatures exceeded 35°C, grasshoppers could achieve body temperatures within the preferred range by moving to the appropriate height on vegetation. These results illustrate the advantage of assessing behavior in the field in relation to preferred body temperatures determined in the laboratory.

## Introduction

Grasshoppers exhibit habitat selection on a variety of spatial and temporal scales (Samways and Sergeev 1997). At the broadest scale, some migrate hundreds of kilometers over the course of one or more weeks in response to changes in habitat quality (Farrow 1990). At a smaller scale, grasshoppers typically move several meters per day, responding to the distribution of food, mates, oviposition sites, and suitable microclimates (Joern 1983; Smith and Grodowitz 1987; With 1994). Other adaptive movements are likely to occur rapidly over spatial scales of several centimeters and intervals of several seconds. Constraints due to microclimatic heterogeneity may force grasshoppers to respond this rapidly if they are to approach or attain preferred body temperatures, and avoid stressful temperatures (Chappell and Whitman 1990). Behavioral responses to temperature must be dynamic, because the location of favorable microhabitats varies during the day with environmental temperatures, the incident angle and intensity of solar radiation, wind speed, humidity, and, perhaps, with varying thermal requirements of the grasshoppers themselves. However, although microclimatic variation may constrain grasshoppers, patchy thermal environments also present them with numerous options for regulating body temperature.

Behavioral thermoregulation was examined in the grasshopper *Melanoplus sanguinipes* (F.) (Orthoptera: Acrididae). Several previous studies of this species examined its behavioral responses to microclimate and recorded body temperatures in both the lab (Lactin and Johnson 1996a, b, 1997b, 1998; Samietz et al. 2005) and field (Chappell 1983; Kemp 1986; O'Neill 1994; O'Neill et al. 1994). However, the short-term dynamics of behavioral thermoregulation of grasshoppers, where responses occur over intervals of less than several minutes, have been little studied in the field. In addition, although previous field studies have documented body temperatures attained by *M. sanguinipes* in the field (e.g. Chappell 1983; Kemp 1986), the effectiveness of thermoregulation has not been evaluated relative to an independent assessment of the preferred body temperature, an approach recommended by Hertz et al. 1993. Our goal, therefore, was to determine if and when behaviors exhibited in the field allowed adult *M. sanguinipes* to achieve body temperatures within their preferred range. First, a laboratory thermal gradient was used to determine the preferred (set-point) body temperature of adults. The short-term behavioral responses of adults to microclimate heterogeneity were then examined in the field. Finally, body temperatures attained by grasshoppers in the field were measured by determining the operative body temperatures of grasshopper models placed in the range of locations, postures, and orientations that had been observed in the field. Whether vegetation structure influenced the ability of grasshoppers to raise body temperature in the morning, and to avoid overheating in the afternoon, was also examined.

## Materials and Methods

### Preferred body temperatures

A laboratory thermal gradient was used to determine preferred body temperatures of adult *M. sanguinipes*. The advantage of measuring preferred body temperatures in the laboratory, rather than in the field is that the grasshoppers are more likely to select body temperatures independently of constraints faced in the field, such as predation and the unsuitability of transitional microhabitats during their movements (Chappell and Whitman 1990; Hertz et al. 1993). The thermal gradient consisted of four parallel 15 × 107 × 0.8 cm thick aluminum bars, the ends of which rested upon two square (in cross-section), 2.5 cm wide, hollow aluminum tubes oriented perpendicular to the long axis of the bars; three of these bars were used in this study. Water from a hot water bath was pumped in a loop through the two tubes beneath one end of the bars, while water from a cold bath was pumped through the tubes at the other end. The surface temperature of bars (T_BAR_) was measured with 0.25 mm diameter thermocouples taped 10–20 cm apart to the center top of each bar. T_BAR_ ranged from 13.8–16.0°C at the cold ends of the three bars to 54.4–55.2°C at the hot ends. In linear regressions of the relationship of location on the bar to T_BAR_, the r2 was 0.99–1.00. Each bar was enclosed within a 3 cm high clear plastic cover that confined the grasshoppers and stabilized temperature; the entire apparatus sat within a larger opaque enclosure. Except during observation, when the bars were illuminated by red light, grasshoppers were in complete darkness to minimize outside disturbance or orientation to an external light source. Prior to introducing grasshoppers to the gradient, we fed them *ad lib* overnight on Romaine lettuce and wheat bran in a 25°C incubator.

A four-step procedure was used to estimate the body temperatures of grasshoppers on the gradient. First, after enclosing 14–18 adult grasshoppers on each bar, the position of all grasshoppers on the gradient was recorded every 60 min for 4 h, each recording period lasting <5 min. Males and females were placed on separate bars so that sexual interactions would not interfere with thermoregulatory responses. The positions of grasshoppers that were stationary on the surface of bars were recorded; but the positions of the few grasshoppers that had moved to the side or top of the clear plastic enclosure were not recorded. Second, regressions of T_BAR_ with distance along the gradient were used to estimate T_BAR_ at the exact position occupied by each grasshopper (beneath the center of its thorax). Third, the body temperature of grasshoppers was estimated at different locations by determining the relationship between T_BAR_ and operative body temperatures (T_E_) of grasshoppers on the gradient. T_E_ is an estimate of the body temperature that an animal achieves when metabolic heat gain and evaporative heat loss are insignificant (Bakken 1992), which is likely the case for non-flying grasshoppers not under thermal stress. T_E_ of dead, dried *M. sanguinipes* placed on the gradients (in the same posture adopted by the live grasshoppers), was measured after inserting a 0.25 mm diameter thermocouple into the center of the thorax of each grasshopper and gluing it in place. Each “model” was placed directly over a thermocouple taped to the gradient and its temperature was recorded after 20 min with the covers in place; the temperature of all models had stabilized by 20 min. This was repeated until each model had been placed at every thermocouple location on the gradient.

Finally, to characterize the preferred body temperature range, the procedure of Hertz et al. (1993) was adopted for calculating the set point range (T_SET_) as the interquartile range of the estimated body temperatures of live grasshoppers on the gradient. Outliers affect the interquartile range less than they would a mean preferred temperature. The use of the central 50% is common (e.g., Christian and Bedford 1995), although alternatives have been used (e.g., 80% by Diaz [1997]); the influence of the choice of ranges on the interpretation of results will be addressed in the discussion. Variance of T_E_ was lowest at the hour 1 observation period (Bartlett's Test of Homogeneity of Variance, P < 0.001), so those data were used to calculate T_SET_. The variance of positions on the gradient increased after the first hour because some of the grasshoppers began to wander, perhaps in search of food.

### Behavioral observations in the field

During June-August 1994–1996, *M. sanguinipes* were observed 14 km south of Three Forks, Montana, U.S.A. (latitude 45° 45′ N, longitude 111° 35′ W; elevation: 1340 m), the same site from which grasshoppers placed on the gradient were collected. The pasture was dominated by crested wheatgrass, *Agropyron cristatum*. (L.) Gaertn. To characterize grasshopper behavior within different vegetative structures, all observations were made on two contiguous, but differentially-grazed plots, each 35 × 35 m (see O'Neill et al. 2003 for site descriptions). From 1993–1996, one plot was intensively grazed in June, so that vegetation was at a low, uniform height. The soil surface between clumps of grass was mostly free of plant litter, so it was more exposed to direct solar radiation. Because the other plot had been left ungrazed after 1992, standing vegetation was dense and tall, and most bare spots between the grass clumps accumulated a dense mat of plant litter. In order to monitor behavior under a greater variety of environmental conditions, observations were alternated between the two plots. Because no differences were found in the types of behavior exhibited by grasshoppers in the two plots, data from ungrazed and grazed areas were combined for purpose of analysis. All observations were conducted when wind speeds were low and when clouds did not obscure the sun.

The observational methods used were similar to those of Anderson et al. (1979), and were designed to induce grasshoppers to change their location in the habitat, and react to the microclimate in their new location. An observer walked slowly through a plot until a stationary grasshopper was located and flushed. After it landed, the grasshopper was observed continuously for 2 min. Steps were taken to reduce the likelihood that the final location of the grasshopper was not a transitional area, or one chosen by the grasshopper for reasons (e.g., for mating, feeding, or predator avoidance) that might conflict, at least temporarily, with thermoregulation. Observations on a particular individual were discarded if the grasshopper 1) moved to a new location during the last 30 seconds of the two-minute period, 2) interacted with another insect, 3) fed, or 4) was apparently disturbed by the observer during the two minutes (i.e., jumped away following any abrupt movement by the observer).

To characterize the thermal environment experienced by each grasshopper, soil surface (T_S_) and air temperatures were measured using Cole-Parmer Digi-Sense® thermometers and 0.25 mm diameter thermocouples shaded at their tips from direct solar radiation. T_S_ was recorded at the end of each two-minute observation, always on the surface of the patch of fully-insolated soil closest to the final position of the grasshopper. Air temperature (T_TX_) was measured at the thoracic height of each grasshopper at its exact positions at the beginning and end of the two-minute period. The grasshopper's initial and final height above the soil surface was recorded, as well as whether the grasshopper experienced full insolation, full shade, or partial shade (i.e., any degree of shading). During the two minute observation, the following data were also recorded 1) the total linear distance, both horizontal and vertical that the grasshopper moved after it alighted following flushing, 2) the number of stops it made before remaining stationary for at least 30 s at the end of the observation, 3) its body posture (crouched, normal, or stilted), and 4) the orientation of its longitudinal axis in the horizontal plane (LAH) and vertical planes (relative to the point on the horizon above which the sun was located). When in a crouched posture, a grasshopper pressed the venter of its thorax and abdomen against the substrate, whereas a stilting grasshopper extended its legs and elevated its thorax and abdomen well above the substrate. A grasshopper perched at 0° LAH faced the position on the horizon directly below the location of the sun, whereas one perched at 180° faced directly away. At 90° or 270°, its longitudinal axis was perpendicular to the sun's radiation, thus maximizing the total body surface area exposed to direct solar radiation; this is referred to as “flanking behavior” by some authors (e.g., Samietz et al. 2005). Lactin and Johnson (1997) determined that direct, but not diffuse radiation had a significant effect on the body temperature of *M. sanguinipes*. All other orientations were recorded as either 45°, 135°, 225°, or 315°. For purpose of analysis, the eight orientations were grouped into three functional categories: 1) ∼0° (0° and 180° orientations = facing towards or away from the sun), 2) ∼90° (90° and 270° = basking orientations), and 3) ∼45° (all others).

Chi-square analyses were used to determine if the frequency distributions of grasshoppers adopting different behaviors varied with microclimatic conditions, as indicated by T_S_ in the full sun (with observations grouped into 5°C categories). The relationship of T_S_ to perch height and the T_TX_ experienced was examined using Kruskal-Wallis analyses. T_S_, rather than time of day, was used in these analyses because it is a good overall summary of microclimate conditions and it is stable relative to T_A_. In addition, because observations were made over a wide range of dates, those taken at the same time of day on two different dates could occur under different environmental conditions (e.g., ambient temperature and the height of the sun above the horizon).

### Estimates of body temperature in the field

To examine the effect of behavior on body temperature and to assess when and how *M. sanguinipes* attain preferred body temperatures (T_SET_) in the field, the T_E_ of *M. sanguinipes* was measured using models in the field. The alternative approach of “grabbing and stabbing” live grasshoppers with thermocouples was not used. Due to the necessary delay in taking a measurement after a grasshopper was flushed and captured, the grab-and-stab method would not allow us to detect slight differences in body temperature of grasshoppers in different postures, orientations, and microhabitats. Models were prepared in the same way as those used on the thermal gradient. Using the behavioral observations as a guide, T_E_ was measured in six microhabitat/orientation combinations that would likely generate the entire the range of possible body temperatures for *M. sanguinipes* in the field on sunny days: 1) in a crouched posture on fully insolated bare soil, with the longitudinal axis oriented at 90° or 270° (= basking orientation, 2) on fully insolated bare soil or a thin layer of matted plant litter, with the longitudinal axis oriented at 0° so that the body was aligned with and partially obscured by the shadow of a standing grass stem, 3) in full sun 2 cm above the soil surface on a horizontal stem or elevated, matted grass, with the longitudinal axis of the body oriented directly at the sun in both the horizontal (0°) and vertical planes, 4) 5 cm above the soil surface in a vertical orientation against the shaded side of a grass stem but with the body only partially shaded, 5) same as 4, but 10 cm high, and 6) same as 4, but 20 cm high. T_E_ was recorded after the temperature of the model stabilized at 38 locations in the grazed plot and 41 in the ungrazed plots. The locations were determined by blindly tossing a 40 cm diameter metal ring into the plot. In some locations, the T_E_ for one of the location/orientation combinations listed above was not recorded because the appropriate microhabitat was unavailable (e.g., there was no 20 cm high grass stem in the sample ring). In total, 510 measurements were made, all under cloudless conditions and at low wind speeds. T_S_ were also recorded at each location, so that T_E_ could be regressed on T_S_. Selected paired comparisons were then made among locations using Wilcoxon Signed-Rank Tests.

**Figure 1.**
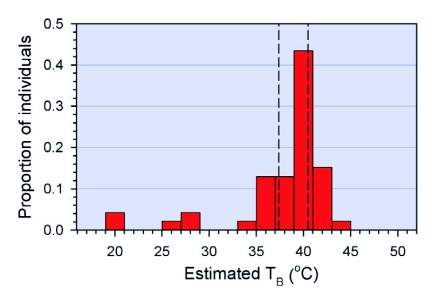
Distribution of adult *M. sanguinipes* on laboratory thermal gradient bars; vertical lines represent limits of set point range.

To determine whether the physical structure of vegetation influenced the ability of *M. sanguinipes* to thermoregulate, T_E_ estimates were compared using another set of models placed in the grazed and ungrazed plots at the Three Forks site, and at a site with a similarly-grazed pair of plots at the Montana State University Red Bluff Research Ranch 3 km east of Norris, Madison, Co., Montana. All measurements were made during cloudless periods and a single model was used in both plots on each day; the locations for the models were determined using the metal ring, as described above. On five days, the models were placed on the soil surface in a basking orientation and posture, alternating measurements between grazed and ungrazed plots. To determine whether basking was more effective in the grazed plots when temperature was relatively low early in the day, ten pairs of measurements made each day (always between 0659 and 1013 h) were combined into a single set of 50 data pairs. To determine whether model temperature was higher on the ground in the grazed plots during the hotter periods of the day, ten pairs of measurements made on each of four days (always between 1148 and 1419h) were combined into a single set of 40 pairs; late measurements were not made on one day because clouds obscured the sun after 1110. On two days, a similar set of observations was conducted, placing models in a vertical position, venter towards the direction of the sun, halfway up the highest grass stem within the metal ring, resulting in 20 pairs of early and 20 pairs of late measurements. Data were analyzed with Wilcoxon Signed Rank Tests.

## Results

### Preferred body temperatures

*Melanoplus sanguinipes* adults had a wide range of potential body temperatures available on the gradients, from 15.9–51.8°C. Because the gradient was linear and the bars were rectangular in shape, all potential body temperatures were equally available, with perhaps a few constraints because more than one grasshopper was on each bar. However, after onehour on the gradient, most grasshoppers aggregated within a relatively narrow band, where T_E_ ranged from 36–42°C, and T_SET_ from 37.4–40.5°C (Figure 1).

### Behavior in the field

In the field, grasshoppers always had both fully-insolated and shaded microhabitats available. When T_S_ was ≤30°C, 75% of the grasshoppers that moved during the two-minute observations moved to a location more insolated than the one to which they were flushed (N = 141, Fig. 2a); only one grasshopper moved to a more shaded location. At these temperatures, flushed grasshoppers that landed in shade or partial shade often moved until they entered a fully insolated patch of soil, where they immediately stopped. When we monitored this in 1995 at T_S_ ≤ 30°C, 42% of 36 grasshoppers responded in this way to the shade-sun transition. As T_S_ increased, the proportion of those moving to a more insolated location decreased, while the proportion moving to shade increased (chi-square contingency table analysis; χ^2^ = 132.3 d.f. = 8, P < 0.001). At T_S_ > 40°C, grasshoppers intersecting the slender shade of a single grass stem while walking on the soil surface sometimes stopped and rotated horizontally to align with the slender band of shade.

**Figure 2.**
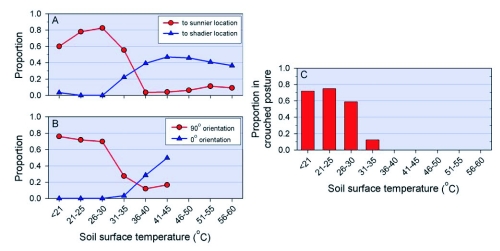
Correlations between behavior and temperature in M. sanguinipes. A) Proportion of grasshoppers moving towards areas of greater or lesser insolation compared to their initial location (*n* ranges from 5–38 among temperature categories); B) Proportion of grasshoppers adopting basking orientation (90°) or facing direction of sun (0°) (*n* ranges from 21–37 for among temperature categories, with the exception of 41–45°C, where *n* = 6); C) Proportion of grasshoppers perched on soil surface that adopted the crouched posture (*n* ranges from 5–34); [continued on next page]

When in full sun or partial shade at low temperatures, individuals on the soil surface tended to perch in a basking orientation (90° or 270°). The proportion of grasshoppers basking varied across temperatures (Figure 2b; χ^2^ = 40.76, d.f. = 5, P < 0.001). After flushing, many grasshoppers that moved to a fully insolated patch immediately rotated horizontally, as if on a turntable, to achieve the basking orientation. Rotation was common at T_S_ ≤ 25°C (81% of 36 grasshoppers on ground in full sun), less common at 26–30°C (50% of 32 grasshoppers), and relatively rare at 31-40°C (11% of 44 grasshoppers; χ^2^ = 38.9, d.f. = 2, P < 0.001). In contrast to those individuals observed at low temperatures, the 76 grasshoppers observed in full sun at T_S_ > 40°C often perched in a manner that minimized intercepted radiation. Of the six orientations recorded in the horizontal plane, one-third (0° and 180°) placed the grasshopper generally facing towards or away from the sun. Of the three orientations recorded in vertical plane, that is approximately horizontal, oblique (∼45°), or vertical relative to the soil surface, the latter two minimized intercepted radiation during times of day when T_S_ was high and the sun was well above the horizon. Thus, if the grasshoppers orientated randomly (among the 18 possible combinations), only 22.2% should have been in orientations that minimized intercepted radiation; at T_S_ > 40°C, however, 48% of 76 individuals adopted one of the four insolation-minimizing orientations (chi-square goodness-of-fit, χ^2^ = 73.4, df = 1, P < 0.001).

The basking orientation was often combined with a crouched posture that brought the grasshopper closer to the soil surface, sometimes with their abdomens laying against the ground. Those in the crouched posture at 90° orientation also extended their legs horizontally on the insolated side of the body with the result that they minimized self-shading; they also raised the hind leg on the shaded side of the body where it received direct radiation; in 1995, 48 of 49 basking grasshoppers adopted this posture. Among adults perched on the soil surface, the proportion crouching decreased as T_S_ increased (Fig. 2c; χ^2^ = 69.32, d.f. = 5, P < 0.0001). Only three individuals on the soil surface, all at T_S_ from 39–46°C, were observed in the stilted posture at their final location. We found no relationship between T_S_ and the height of the surfaces to which grasshoppers were flushed (initial perch height) (Kruskal-Wallis Test, P = 0.29), suggesting that grasshoppers do not control jumps to reach a particular thermal environment. However, final perch height after two minutes increased with T_S_ (Figure 2d) (P < 0.001). None of the 49 grasshoppers observed at T_S_ > 50°C remained on the soil surface.

**Figure 2.**
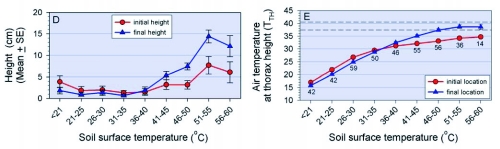
[continued] D) mean height of grasshoppers at initial and final locations (sample sizes same as in E); E) Air temperature at thorax height (T_TX_) at the initial and final location of the grasshopper (numbers below symbols are sample sizes for each temperature interval); for reference, the pair of horizontal dashed lines represent the bounds of the T_SET_ range measured in the laboratory.

By monitoring the T_TX_ of grasshoppers at the beginning and end of each observation period, it could be determined whether they moved to areas of higher or lower air temperature. A clear relationship was observed between T_S_ and the temperature differential (Figure 2e) (Kruskall-Wallis Test, P < 0.001). Among the 145 grasshoppers observed at T_S_ ≤ 30°C, 70% moved towards higher T_TX_ (maximum = 7.8°C increase), whereas only 4% moved to lower T_TX_ (maximum = 3.8°C decrease). But of the 142 grasshoppers observed at T_S_ > 40°C, 85% moved towards lower T_TX_, usually by moving up onto vegetation (maximum = 17.3°C decrease), and none moved to a location with a higher T_TX_.

To roughly estimate the amount of time and effort a grasshopper invested in locating a suitable microclimate during the two minutes following flushing, the relationship of T_S_ to the total distance each grasshopper traveled, and the number of times it stopped before settling in one location were examined. Distances tended to be shorter at intermediate T_S_, because only 22% of the grasshoppers relocated after flushing at T_S_ from 36–40°C, whereas 73% moved at all other temperatures combined. However, in a quadratic regression (distance = 58.78 - 2.98(T_S_)^2^ + 0.049; F_2,397_ = 24.1, P < 0.001), T_S_ explained just 11% of the variation in distance moved by adults. Similarly, the number of times that a grasshopper halted increased with T_S_ (number stops = 0.012T_S_ + 1.69; F_1,359_ = 5.89, P = 0.02), but T_S_ explained just 1% of the variation.

During the study, several observations were made that, though not fully quantified, were seen often enough to suggest that they are common and often subtle aspects of thermoregulatory behavior. While presenting their flanks to the sun, many basking grasshoppers, for example, also tilted their dorsoventral axes (i.e., leaned) away from the direction of the sun. This behavior was also observed in basking robber flies at the same location and was interpreted as a means for them to maximize intercepted radiation by making the “plane” of their lateral surface as close as possible to being perpendicular to incoming radiation (O'Neill et al. 1990). In addition, before basking grasshoppers settled into place, they often moved laterally a short distance to place their bodies against the insolated side of a plant or a depression in the soil, which may have reduced convective heat loss or placed them in a warmer microhabitat. Occurrences of this were recorded eight times in 1995, all at soil surface temperatures of 16–30°C. Other behaviors were exhibited only when T_S_ was high. When flushed to a hot soil surface, some grasshoppers either 1) walked in the stilted postures before climbing up or hopping to vegetation (recorded four times, only at T_S_ of 43–50°C) or 2) immediately hopped up onto vegetation after no more than one second on the ground (recorded 10 times, all at T_S_ of 48–54°C).

**Figure 3.**
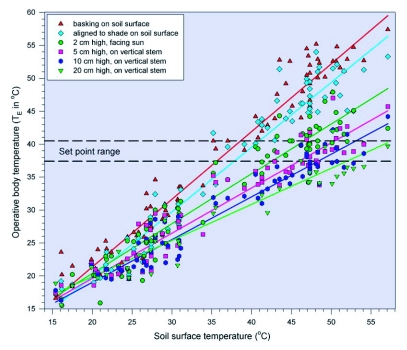
Operative body temperature (T_E_ in °C) of grasshopper models placed at different heights and orientations; pair of horizontal dashed lines represents the T_SET_ range measured laboratory.

**Table 1.**
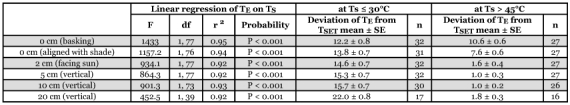
Regression of model grasshopper temperatures (T_E_) on soil surface temperature (T_S_) in field, and deviation of T_E_ from T_SET_ for models placed in different combinations of location and posture.

### Estimated body temperatures in the field

Grasshopper models were used to address several questions concerning the value of behavioral thermoregulation at different times in different habitats. First, to what extent can behavior modify body temperature? For all microhabitat/orientation combinations, T_E_ increased linearly with T_S_ (Table 1, Figure 3). Paired comparisons among models indicated that *M. sanguinipes* can adjust body temperature by making relatively minor behavioral changes throughout the day (Table 2), raising temperature early in the day, but minimizing it later in the day relative to possible alternatives.

**Figure 4.**
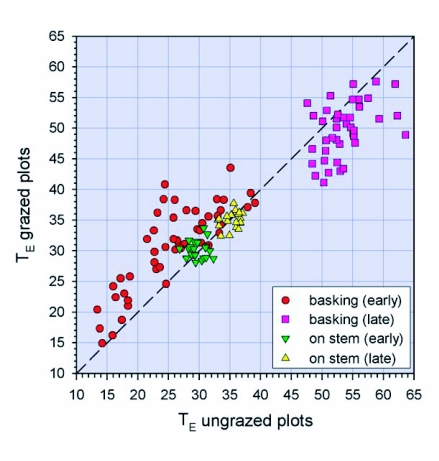
Comparison of operative body temperature (T_E_ in °C) in grazed and ungrazed areas for grasshopper models placed in basking orientation and posture or halfway up the tallest grass stem at the sampling location; dashed line indicates equality of temperature in the two plots.

Second, how closely does T_E_ approach T_SET_ for models simulating different grasshopper behaviors, and how does this vary with environmental temperature? At T_S_ < 30°C, all models had temperatures below the lower T_SET_ limit (Figure 3). Those in different posture/height combinations deviated from T_SET_ by averages of 12–22°C (Table 1). At T_S_ > 45°C, none of models on the soil surface were within T_SET_. However, 31.3% of the models placed at heights of 2–20 cm were within T_SET_, and the mean deviation from T_SET_ was always <2°C.

**Table 2.**
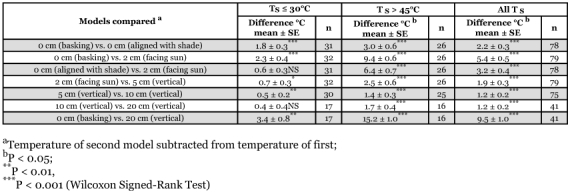
Selected paired comparisons of T_E_ between models placed in different combinations of location and posture.

Third, with model T_E_ data as a basis for prediction, how often were live grasshoppers observed within T_SET_? Because our observations were used as a guide to placing the model grasshoppers, many of the live grasshoppers observed had locations, postures, and orientations that closely matched those of models. For live, basking grasshoppers in full sun at 0 cm (N = 54) and for those perched vertically on stems from 2–20 cm (N = 56), the regressions of T_E_ on T_S_ were used to estimate the T_B_ of the live grasshopper, which was then compared to T_SET_ and T_TX_ at each grasshopper's final location. For those perched on stems, interpolation was used to estimate T_B_. T_B_ of basking grasshoppers, all observed at T_S_ ≤ 26°C, were estimated to be 1.7 ± 0.2°C in excess of T_TX_ (maximum = 4.8°C), but none of the basking grasshoppers had an estimated T_B_ within T_SET_ (mean deviation = 16.6 ± 0.3°C). Grasshoppers on the stems, all observed at T_S_ > 40°C, were estimated to be 6.1 ± 0.4°C in excess of T_TX_ (maximum = 12.5°C), and 43% of live grasshoppers on stems had estimated T_B_ within T_SET_. Those below T_SET_ deviated from the lower bound by 3.4 ± 1.0°C (N = 24), whereas those above T_SET_ deviated from the upper bound by 1.4 ± 0.4°C (N = 8).

Fourth, do differences in vegetation structure due to grazing influence the ability of grasshoppers to thermoregulate? Comparisons of models in the grazed and ungrazed plots indicate that grazed vegetation allowed *M. sanguinipes* to bask more effectively. Early in the day, 82% of the 50 “basking” models were fully insolated in the grazed plots, compared to 16% in the ungrazed plots (χ^2^ = 41.0, d.f. = 1, P < 0.001). As a result, basking models in the grazed plots had mean T_E_ that was 5.0 ± 0.6°C higher than in the ungrazed plots (Wilcoxon Signed Rank Test, P < 0.001) (Figure 4). The deviation of T_E_ from T_SET_ was 7.1 ± 0.9°C for grazed plots (12% within T_SET_) and 11.7 ± 1.9°C for ungrazed plots (4% within T_SET_) (Wilcoxon Signed-Rank Test, P < 0.001). During hotter periods, when most of the 40 basking models on the ground were fully insolated (100% in the grazed plots, 95% in the ungrazed plots), models in the grazed plots had mean T_E_ that was 3.8 ± 0.7°C lower than in the ungrazed plots (N = 40 in each plot; Wilcoxon Signed Rank Test, P < 0.001). However, all models were in excess of the upper limit of T_SET_, the deviations being 9.3 ± 0.7°C for grazed plots and 13.1 ± 0.6°C for ungrazed plots (Wilcoxon Signed-Rank Test, P < 0.001). Models placed halfway up the tallest stem at each sampling point were at 16.6 ± 0.8 cm in the grazed plot and 26.9 ± 0.9 cm in the ungrazed plot (Mann-Whitney Test, P < 0.001). For these models (Figure 4), however, there were no differences in T_E_ between the two plots either early in the day (i.e., before 1100 h; Wilcoxon Signed Rank Test, P = 0.37; N = 20) or later in the day (i.e., after 1230 h; P = 0.28; N = 20). Thus, although grasshoppers in ungrazed plots had more options for vertical movement, different vegetation structures appear to be more constraining to grasshoppers on or near the soil surface, than to those perched on vegetation.

## Discussion

Within their preferred temperature ranges, grasshoppers probably maximize physiological performance and optimize growth rate (Chappell and Whitman 1990, Samietz et al. 2005). Well below the preferred range, grasshoppers are constrained by minimum body temperature thresholds for activity. The minimum temperatures for walking are about 12°C for *M. sanguinipes* ( = *M. mexicanus*; Parker 1930) and 11°C for *Taeniopoda eques* (Whitman 1988). As temperatures rise above the minimum threshold, rates of development, walking, feeding, and defecation in *T. eques* all increase with temperature, as do the times required to pass a spermatophore, deposit eggs, and mate (Whitman 1988). *M. sanguinipes* also exhibits temperature-dependent feeding rates (Lactin and Johnson 1995) and metabolic rates (Chappell 1983), and probably faces most of the rate constraints faced by *T. eques*. In both species, the metabolic advantage afforded by increasing temperature is eventually offset by thermal stresses. The critical thermal maximum has been estimated as 53°C for *M. sanguinipes* (Parker, 1930), but chronic problems related to high temperature, such as increased rates of desiccation, probably occur at temperatures below the critical thermal maxima.

Chappell and Whitman (1990) suggest that preferred body temperatures of acridid grasshoppers range from 30–44°C, though further work on laboratory thermal gradients is likely to refine ranges for individual species, sexes, and developmental stages. Using the method outlined by Hertz et al. (1993), an estimate was obtained of the preferred body temperatures of *M. sanguinipes* to be used as a criterion for judging the potential for effective behavioral thermoregulation in the field. The distribution of estimated body temperatures on the gradient was unimodal, peaking near 40°C.

The 37–41°C T_SET_ range determined for adults was nearly identical to the T_SET_ estimates for *M. sanguinipes* nymphal instars I–V on the same gradient (Rolston 1997), suggesting that there are no major developmental changes in T_SET_ in this species. Lactin and Johnson (1996a), after placing *M. sanguinipes* nymphs on temperature gradients established with incandescent bulbs and using a different method of calculation, estimated the preferred range to be 35–43°C; both their estimate and ours have the same median value of 39°C.

In the field, *M. sanguinipes* adults progressed through a consistent daily sequence of often subtle behavioral responses within their heterogeneous thermal environments. Early in the morning, they quickly moved to warmer, insolated patches of soil surface, which often placed them in shallow depressions in the soil or against the bases of plants. Here they usually rotated to orient the long axes of their bodies perpendicular to the direction of the sun, often while crouching and positioning their legs in a manner that placed them against the soil surface and minimized self-shading. Together, these behaviors maximized body temperature within the available range by maximizing the amount of direct solar radiation intercepted (Lactin and Johnson 1997), and minimizing convective heat loss. For small animals, convective heat loss is lowest near the soil surface and against large objects (Stevenson 1985). Later in the day, *M. sanguinipes* avoided high body temperatures by seeking cooler, often shadier locations and by occupying perches increasingly higher above the soil surface. In both this and a previous study (O'Neill et al. 1994), grasshoppers immediately hopped to plants when they came in contact with soil surfaces with temperatures in excess of 55°C. The repertoire of thermoregulatory behaviors that we observed, including basking (flanking), crouched and stilted postures, seeking shade, and movement in the vertical temperature profile, have also been observed in other grasshoppers (e.g., Waloff 1963; Anderson et al. 1979; Chappell 1983; Gillis and Smeigh 1987; Whitman 1987; Samietz et al. 2005). Our analysis of T_E_ of model grasshoppers and estimates of the T_E_ of live grasshoppers indicate that *M. sanguinipes* adults can use these behaviors to attain temperatures well in excess of ambient and that they were frequently within T_SET_ during hotter times of day.

Several other studies provide independent evidence that *M. sanguinipes* stabilizes body temperatures in the field near the T_SET_ estimated in the laboratory in our study. Working at the same site at which we conducted our study, Kemp (1986) measured body temperatures of living adult *M. sanguinipes* across a wide range of ambient temperatures, regressing body temperature on ambient using a logistic model. Body temperatures rose during the day, but eventually stabilized at about 40°C for males and 41°C for females, at or near the upper limit of preferred range of temperatures we measured on the gradient. In a population in California, *M. sanguinipes* body temperatures peaked in the range centered around 40°C (Chappell 1983). The coincidence between our T_SET_ estimate, the field body temperatures measured by Kemp and Chappell, as well as the range of behaviors and T_E_ that we measured in the field, suggest that *M. sanguinipes* can achieve preferred temperatures at a wide range of ambient air temperatures. In our study, however, grasshoppers were able to do that only after T_S_ exceeded ∼35°C. Similarly, the majority of the *M. sanguinipes* observed by Chappell (1983), including all grasshoppers captured before ∼0930 h and after ∼1630 h, did not have T_B_ within the T_SET_ we determined; the relevance of the this depends, of course, on whether T_SET_ is similar in the two populations.

The fact that many grasshoppers in our study still had estimated body temperatures outside of the T_SET_ range has several possible explanations that are not mutually exclusive. First, perhaps the decline in physiological performance and increase in thermal stress experienced as a grasshopper's body temperature deviates from optimum temperature is more gradual than reflected in the use of the interquartile range. If so, a broader range may be a more appropriate measure of T_SET_. This problem can only be addressed by further research on the relationship of temperature to physiological performance and thermal stress. Second, under field conditions there may be more inter-individual variation in preferred body temperatures, related, for example, to the need to combat infection through behavioral fever (Boorstein and Ewald 1987) or the need to conserve water. Third, the thermoregulatory options perceived by a grasshopper may be different in the field compared to the gradient. A grasshopper on the gradient is faced with a smooth continuum of temperatures leading to the preferred optimum. In the more thermally heterogeneous field environment, however, a grasshopper attempting to walk from a location where body temperature is near optimum may be immediately faced with a location where it deviates even farther from optimum and may become stressful. For example, a grasshopper perched on a short stem where T_E_ = 32°C may have to traverse a soil surface where T_E_ = 57°C when attempting to move to a location where T_E_ is within T_SET_. Thus, staying in place may be the best option in the short-term if the grasshopper cannot reliably predict where the nearest prime thermal microhabitat is located or is unable to move to it without being thermally stressed. Fourth, grasshoppers were observed only over a two-minute interval, during which time they settled into place and into apparent thermoregulatory postures and orientations. However, it is possible that, over longer intervals, they further refine their behavior to come closer to the preferred optimum.

Based on T_E_ estimates, *M. sanguinipes* at our site had body temperatures as much as 12.5°C in excess of air temperature measured at the exact location of the grasshopper's thorax. Chappell (1983) commonly found that live *M. sanguinipes* achieved body temperatures 15-20°C in excess of ambient. In a review of literature, Chappell and Whitman (1990) reported that maximum temperature differences between air and body temperatures for free-living grasshoppers ranges from 7-18°C (the high value being for *M. sanguinipes*). However, examination of the methodology used in different studies reveals some inconsistency in where “ambient” or “air” temperature was measured relative to the position of the grasshopper; and some papers do not say exactly where air temperature was measured. Body temperature has sometimes been compared to air temperature measured at a location away from the grasshopper, in a different, often cooler, microclimate (e.g., at a different height and in the shade). To assess how far a grasshopper can raise body temperature above air temperature using a particular behavior, a comparison of the body temperature of a grasshopper basking on the soil surface to air temperature one meter above the ground in the shade is not as relevant as a comparison to air temperature at the grasshopper's exact location (our approach, and that of others, for example Chappell 1983). Because air temperatures decline rapidly in the first 10 cm above the soil surface, measuring air temperature at some constant height well above where grasshoppers are located can lead to overestimates in the temperature excesses afforded by behavioral tactics.

Although *M. sanguinipes* has the option to raise body temperature early in the day and attain preferred temperatures later, thermoregulation may come at a cost. Jech (1996) reported that six species of grasshoppers spent 37–82% of their time from “daybreak to dusk” basking, but just 4–32% feeding. A stationary basking grasshopper or one that climbs a grass stem to increase convective heat loss is limited in its ability to locate food or mates, and may be more vulnerable to predators. However, *M. sanguinipes* do have some options to avoid stressful high temperatures while still remaining active for short periods. For example, while scavenging livestock dung, *M. sanguinipes* sat and fed within shade of cavities in the dung, where T_E_ averaged 17–5°C below locations on nearby bare, insolated soil surfaces (O'Neill 1994). Similarly, while scavenging the dead bodies of other grasshoppers when T_S_ exceeded 45°C, *M. sanguinipes* commonly fed while standing in a stilted posture atop the cadaver or after dragging the cadaver up onto a plant stem (O'Neill et al. 1994); stilting results in a significant reduction in body temperatures relative to grasshoppers in normal or crouched postures (Chappell 1983; O'Neill et al. 1994).

Within a field, grasshopper species may be unevenly distributed among different microhabitats (Kemp et al. 2002). Though likely linked to variation in food plant preferences, this could also be related to variation in thermal environments among patches and to variation in thermoregulatory strategies among species. For example, on shortgrass prairie, *Psoloessa delicatula* occupies open vegetation and exhibits behaviors similar to those of *M. sanguinipes. Eritettix simplex*, in contrast, is restricted to dense vegetation with greater shading and has a more limited repertoire of thermoregulatory strategies; its greater susceptibility to desiccation apparently precludes its exploitation of open habitats (Anderson et al. 1979). Coxwell and Bock (1995) found *Aeropedellus clavatus* more abundant on east-facing slopes where it achieved higher body temperatures and higher growth rates. Differences in thermal tolerance and thermoregulatory abilities have also been implicated in the differences in the distribution of four grasshopper species among sites with different vegetation heights in Great Britain (Willott 1997). Together, these results suggest that the physical structure of vegetation interacts with the thermoregulatory behaviors of grasshoppers to determine the potential range of body temperatures achieved and, perhaps, to influence distribution of species within a landscape. *M. sanguinipes* is one of the most widely distributed grasshoppers in North America (Capinera et al. 2004), as well as across different vegetation communities and elevations in our area (Kemp et al. 1990; Wachter et al. 1998). Samietz et al. (2005) collected *M. sanguinipes* from altitudes as high as 2650 m in California, and found that *M. sanguinipes* from the highest altitudes exhibited the greatest tendency to use behavior to elevate body temperature, probably as a means of accelerating development in cool environments with short growing seasons.

At the sites used in this study, comparison of T_E_ of “basking” models in grazed and ungrazed plots suggests that bare, unshaded soil in the grazed plots provided superior basking sites during cooler times of day, while being less stressful during hotter times of day. The latter may be due to greater wind speeds near the soil surface in short, sparse grazed vegetation compared to ungrazed plots (O'Neill et al. 2003). A similar comparison for models “perched” halfway up the tallest stem at a sampling point revealed no differences either early or late in the day. Although the grazed plots tended to have higher temperatures near the soil surface, winds speeds (and therefore the potential for convective heat loss) were also higher in the canopy. Despite this, *M. sanguinipes* tended to be more abundant on ungrazed plots at the site (O'Neill et al. 2003), so perhaps any thermoregulatory advantages were offset by the greater quality or quantity of food available in ungrazed habitat.

The results presented here refine our knowledge of the behavioral responses of *M. sanguinipes* across changing temperatures, which, depending on the time of day, are effective in allowing the grasshoppers to approach or attain preferred body temperatures, and avoid overheating. The results confirm those of other studies suggesting that the ability of the grasshoppers to modify their body temperatures is constrained by the physical structure of vegetation in their habitats. Finally, the approach taken illustrates the advantage of assessing behavior in the field in relation to preferred body temperatures determined in a simple laboratory environment (Hertz et al. 1993) and to air temperatures measured at a location in the environment where each grasshopper is evaluating ambient conditions.

## Abbreviations

LAH: longitudinal axis of grasshopper in horizontal plane
T_B_: body temperature
T_BAR_: surface temperature of bar on laboratory thermal gradient
T_E_: operative body temperature
Ts: soil surface temperature
T_SET_: set point range (interquartile range of the body temperatures of grasshoppers on the gradient)
T_TX_: air temperature at thorax height
